# Functional characterization of furin-mediated lipoprotein lipase cleavage

**DOI:** 10.1242/dmm.052897

**Published:** 2026-07-06

**Authors:** Ming Jing Wu, Chelsea Yang, Sirui Wu, Garth Devlin, Feng-Chang Lin, Aravind Asokan, Saskia B. Neher

**Affiliations:** ^1^ Department of Biochemistry and Biophysics, The University of North Carolina Chapel Hill, Chapel Hill, North Carolina 27599, USA; ^2^ Department of Biostatistics, UNC Gillings School of Global Health, Chapel Hill, NC 2759, USA; ^3^ Department of Surgery, Duke University School of Medicine, Durham, NC 27710, USA

**Keywords:** Lipoprotein lipase, Furin protease, Gene therapy, Familial lipoprotein lipase deficiency, Hypertriglyceridemia

## Abstract

Lipoprotein lipase (LPL) is the rate-limiting enzyme that hydrolyzes triglycerides within circulating lipoproteins. LPL dysfunction leads to familial LPL deficiency, which is characterized by chylomicronemia and high risk for acute pancreatitis. Although cell culture studies indicate that the protease furin inactivates LPL by cleavage, the physiological relevance of this process remains unclear. In this study, we investigated the impact of furin-mediated LPL cleavage *in vivo* using inducible knockout mouse models and gene therapy. After identifying the tissue-specific prevalence of LPL cleavage, we compared mice expressing a furin-resistant LPL mutant versus furin-sensitive LPL. Our results demonstrate that furin-resistant LPL lowers longitudinal plasma triglyceride levels without causing adverse effects, such as hepatic steatosis. These findings highlight that engineered furin resistance is a viable strategy to enhance the metabolic function of LPL.

## Teaser

**Summary:** Familial LPL deficiency is a rare but serious disease. This study highlights the need to develop improved LPL variants for future gene therapy options.

## INTRODUCTION

Familial LPL deficiency (LPLD) is a rare autosomal recessive disease, which is present in only 1–10 individuals per million in the general population (Dron and Hegele, 2020). LPLD is caused by mutations in the *LPL* gene, which encodes lipoprotein lipase (LPL), a secreted enzyme that breaks down the triglycerides found in chylomicrons and very low-density lipoprotein (VLDL). Individuals with LPLD suffer from a severe accumulation of chylomicrons in their plasma. As a result, affected individuals can suffer from eruptive xanthomas, lipemia retinalis and recurrent severe pancreatitis, which can be fatal.

Gene therapy using a C-terminal, two-amino-acid-truncation variant of LPL (known as and hereafter referred to as LPL^S447X^) was approved in 2012 by the European Medicines Agency to treat LPLD complicated by severe pancreatitis. This product AAV1-LPL^S447X^ (alipogene tiparvovec; brand name Glybera) (Gaudet et al., 2010) comprises an adeno-associated virus (AAV) vector of serotype 1 encoding LPL^S447X^. AAV1-LPL^S447X^ was the first commercially approved gene therapy product in the Western world. Although treatment reduced patient triglyceride levels in the weeks following administration, these levels returned to baseline within 16–26 weeks (Stroes et al., 2008; Gaudet et al., 2013). Nevertheless, other measures of efficacy have been observed. In an open-label dose-escalation trial, 9 of 14 patients reported improved dietary tolerance and abdominal comfort (Gaudet et al., 2013). Additionally, there was a five-fold decrease in the incidence of pancreatitis during the two-year follow up in this study (Gaudet et al., 2013).

In 2017, uniQure, which marketed Glybera, announced that it would not renew approval of marketing authorization for Glybera (Senior, 2017). Because Glybera is no longer commercially available, individuals with familial LPLD have an unmet need for an LPL replacement therapeutic. An ideal LPL replacement therapeutic would not only reduce pancreatitis but would also reduce plasma triglyceride levels over the long term. Many advances in gene delivery through AAV have been made since Glybera was discontinued, with a focus on making more efficacious AAV gene therapy vectors (Mehta et al., 2023).

One strategy for improving replacement therapies, regardless of the mode of delivery, is to improve the stability and function of the gene product. Most enzymes used in enzyme replacement therapy (ERT) are modified to enhance their half-life, potency and resistance to degradation. For example, the clotting factors used to treat hemophilia are modified with crystallizable fragment fusion technology or PEGylation to enhance their half-life, which reduces the frequency at which patients need to be treated (Peters and Harris, 2018). Replacement therapy for LPL deficiency – through any mode of delivery – could significantly benefit from new and improved LPL variants. Potential improvements to LPL include increasing its resistance to its endogenous inhibitors, reducing its sensitivity to proteases and enhancements to its function.

LPL is susceptible to cleavage by the protease furin (FURIN) (Jin et al., 2005). Furin is an endoprotease belonging to the proprotein convertase family of enzymes. Furin cleaves at the consensus motif R-X-(K/R)-R↓, where X is any residue and ↓ denotes the cleavage site. Human LPL contains the motif RAKR at residues 294–297 (Jin et al., 2005). This cleavage site is located between the N-terminal catalytic domain and the C-terminal domain of LPL, which plays a role binding to lipoprotein substrates (Lookene et al., 1997). Additionally, residues in the C-terminal domain of LPL bind to lipoprotein receptors, such as LRP, to enhance receptor-mediated uptake of remnant lipoprotein particles (Chappell et al., 1993; Beisiegel et al., 1991; Hayne et al., 2017). Furin separates these two domains and renders the N-terminal domain catalytically inactive. There is no established role for the isolated C-terminal domain *in vivo* but, when added to cell culture, this presence of this domain was sufficient to enhance uptake of VLDL particles (Chappell et al., 1994).

LPL activity is downregulated by several macromolecular inhibitors. These include ANGPTL3, ANGPTL4, ANGPTL8 and APOC3 (Zhang, 2016; Kersten, 2014). One potential strategy to boost the potency of an LPL replacement therapy would be to utilize a version of LPL that is resistant to these inhibitors. Although the mechanisms by which ANGPTL3, ANGPTL4 and ANGPTL8 inhibit LPL are not universally agreed upon (Sukonina et al., 2006; Lafferty et al., 2013), some studies report that ANGPTL3 and ANGPTL4 enhance furin-mediated cleavage of LPL. Specifically, Liu et al. report that cultured cells exogenously expressing both ANGPTL3 and LPL show more LPL cleavage product and less full-length LPL (hereafter FL LPL) compared with cells expressing only LPL (Liu et al., 2010). Additionally, Dijk et al. report decreased furin-mediated cleavage of LPL in adipocytes from *Angptl4*^−/−^ mice relative to wild-type (WT) mice (Dijk et al., 2018).

Though it is known that LPL is cleaved by furin, the role that furin-mediated cleavage plays in the regulation of triglyceride levels *in vivo* is not fully understood. The Human Protein Atlas (2024) shows that furin is highly expressed in a wide range of tissues and is also highly expressed in many tissue culture cell lines (Uhlen et al., 2015; https://www.proteinatlas.org/ENSG00000140564-FURIN/tissue). However, the tissues that express LPL, specifically heart muscle, skeletal muscle and adipose tissue, have lower furin expression levels (Uhlen et al., 2015). Most studies used cultured cells, where furin-mediated cleavage drastically reduces the amount of active LPL that is secreted into medium (Wu et al., 2018; Liu et al., 2010; Jin et al., 2021). One study used samples from both humans and mice, focussing on adipose tissue, found that both furin-cleaved and FL LPL are present (Dijk et al., 2018). However, a comprehensive, *in vivo* analysis of the different forms of LPL in different tissues has not been undertaken. Thus, the importance of furin-mediated cleavage on LPL levels or activity *in vivo* has not been fully explored. Here, we tested the prevalence of furin-mediated cleavage of LPL *in vivo.* We also tested the importance of furin-mediated cleavage regarding triglyceride levels by comparing LPL replacement. We did this by using either WT LPL or a mutant variant of LPL resistant to furin-mediated cleavage.

## RESULTS

### Prevalence of furin-mediated cleavage *in vivo*

**Fig. 1.**
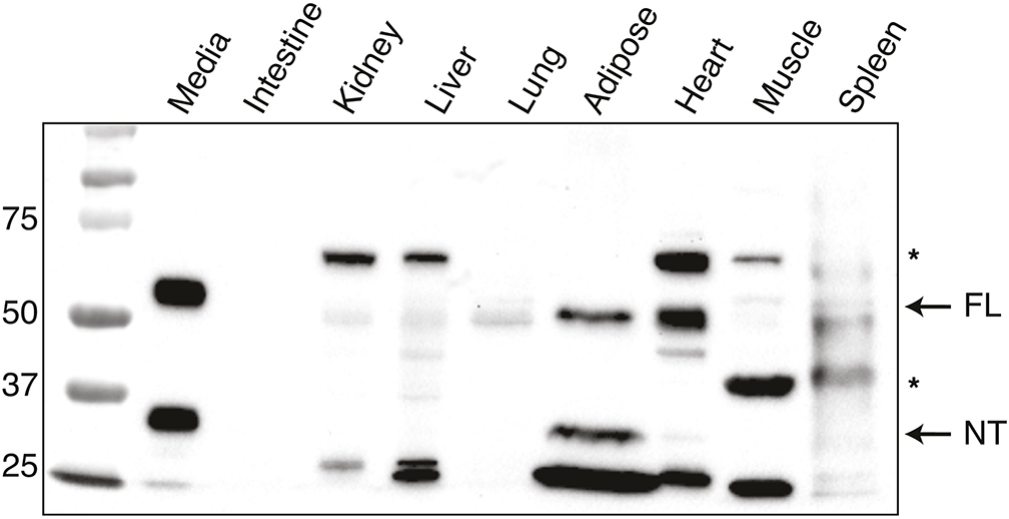
**Forms of endogenous LPL present in tissue.** We analyzed endogenous LPL in different tissues from C57BL/6 mice by western blotting. LPL present in conditioned medium of cultured cells was used as control for the position of full-length (FL) LPL and N-terminal (NT) LPL fragments (arrows). Some tissues have unknown bands present in some tissues, which might indicate cross-reaction or bands or LPL cleavage products of unknown origins (indicated by asterisks). LPL expression was highest in heart and adipose tissue, consistent with published RNA expression data.

Although studies of furin-mediated cleavage of LPL in tissue culture are common due to the high levels of furin produced by cultured cells, we do not know how common furin-mediated cleavage of LPL is in different tissues in the body (Uhlen et al., 2015; The Human Protein Atlas, 2024). We, therefore, performed western blots on several different types of mouse tissue to determine if FL LPL or cleaved LPL was more prevalent. To do so, we used a goat polyclonal antibody against LPL that recognizes both FL LPL and its cleavage fragments. C57BL/6 mice, a common laboratory strain, were used for this experiment. We used LPL produced in cultured cells, which produce FL LPL as well as FL cleavage fragments, as a marker for LPL derived from tissues. As shown in Fig. 1, FL LPL is a polypeptide of 50 kDa. Furin-mediated cleavage yielded an N-terminal (NT) fragment of 32 kDa and a C-terminal (CT) fragment of 18 kDa. The small CT fragment is not always well-resolved on gels and, therefore, cleavage is visualized here by the presence of the larger NT fragment. We probed for LPL in intestines, kidney, liver, adipose, heart, muscle and spleen tissues isolated from 10-month-old C57BL/6 mice. With a molecular mass of 50 kDa, FL LPL, is easily observed in both heart and adipose tissue, consistent with information available at The Human Protein Atlas, which shows the highest levels of LPL mRNA expression in adipose and heart muscle (Uhlen et al., 2015; The Human Protein Atlas, 2024). A faint band for FL was also observed in samples of kidney, liver, lung and muscle tissue (Fig. 1). Adipose tissue and liver tissue also contained NT fragments of slightly <37 kDa (Fig. 1). As can be seen in Fig. 1, each tissue type had additional background bands that did not correspond to a known LPL fragment.

### Treatment of mice with a non-cleavable LPL variant

**Fig. 2.**
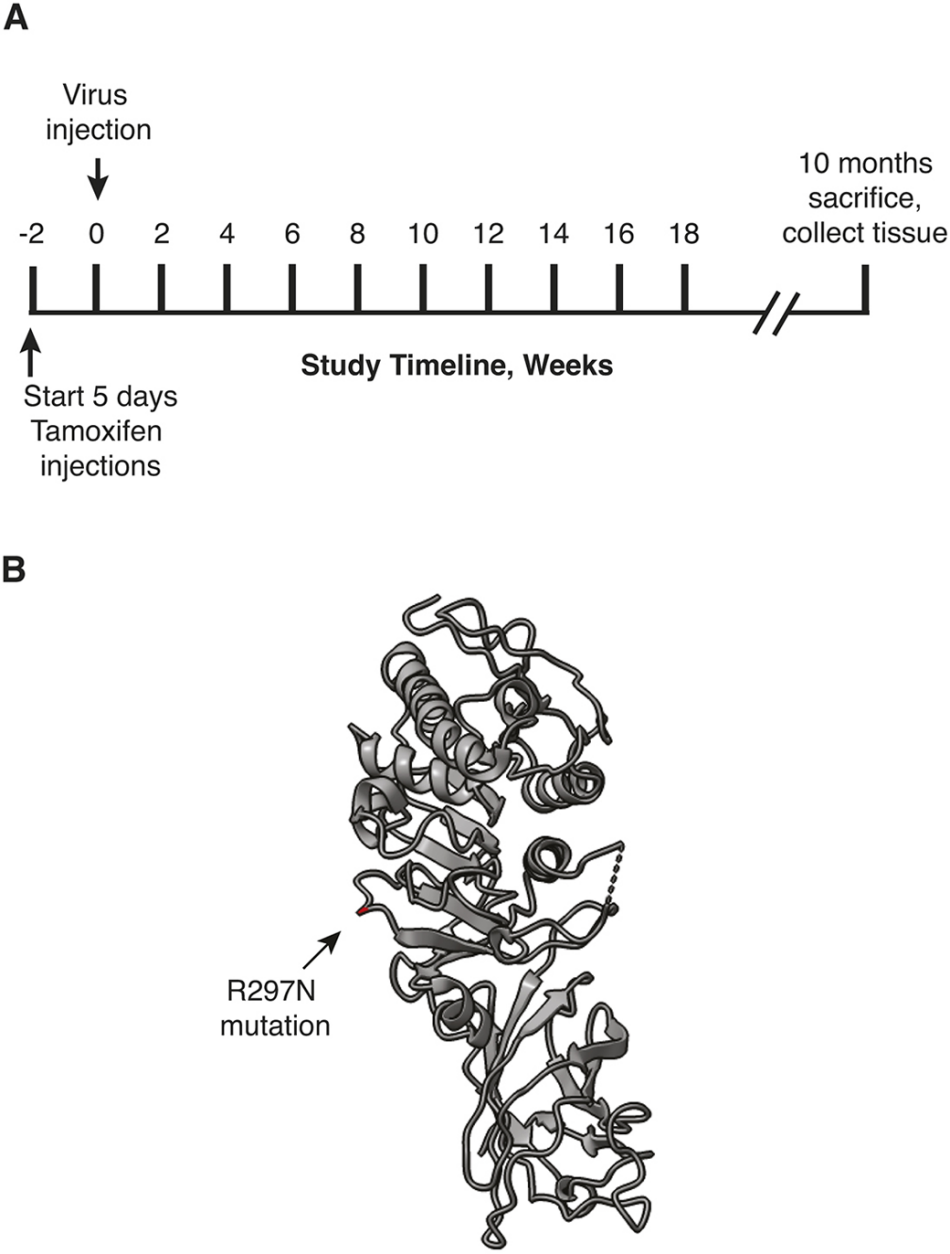
**Study overview.** Two weeks before virus injection (−2) LPL knockout was induced by treatment with tamoxifen for 5 days in 8-week-old whole-body-inducible LPL knockout (iLpl^−/−^) mice. At week 0, plasma triglyceride levels were checked to confirm the loss of LPL, and mice were treated with LPL_WT_, LPL_R297N_ or luciferase (negative control) via adeno-associated virus serotype 9 (AAV9)-mediated gene therapy. Blood was collected every two weeks up to week 18. Mice were sacrificed at 10 months, and their blood and tissue collected. (B) Three-dimensional structure of LPL. Indicated is the position of the R297N substitution as deposited at the Research Collaboratory for Structural Bioinformatics Protein Data Bank (RCSB PDB), PDBID: 8ERL.

We next set out to compare furin-resistant LPL and WT LPL in a mouse model of familial LPL deficiency. This mouse model is a whole-body inducible LPL knockout (iLpl^−/−^), which has been described previously (Noh et al., 2006; Gordts et al., 2016). An outline of the experimental scheme is shown in Fig. 2. Briefly, LPL deficiency was induced in 8-week-old mice with tamoxifen. After 2 weeks, plasma triglyceride levels were checked and LPL variants were added back via adeno-associated virus serotype 9 (AAV9)-mediated gene transfer. The AAV9 serotype was selected because it shows tropism for cardiac and musculoskeletal transduction (Bish et al., 2008; Sun et al., 2008). We tested two LPL variants, both of which were based on the human LPL sequence containing the LPL_S447X_ truncation. This two-residue C-terminal truncation was shown to be effective in rescuing LPL-deficient mice via AAV9-mediated gene therapy whereas FL LPL was not (Ross et al., 2005). By using LPL_S447X_ as a base, we compared LPL that was otherwise wild type (LPL_WT_) and a furin-resistant variant of LPL (LPL_R297N_). LPL_R297N_ is a mutation in the furin recognition motif RAKR of LPL (Fig. 2B), altering this motif to RAKN that we have previously shown to render LPL resistant to furin-mediated cleavage (Wu et al., 2018). A cohort of iLpl^−/−^ mice were also treated with the gene for a luciferase (firefly luciferase) delivered via AAV9 as a negative control.

**Fig. 3.**
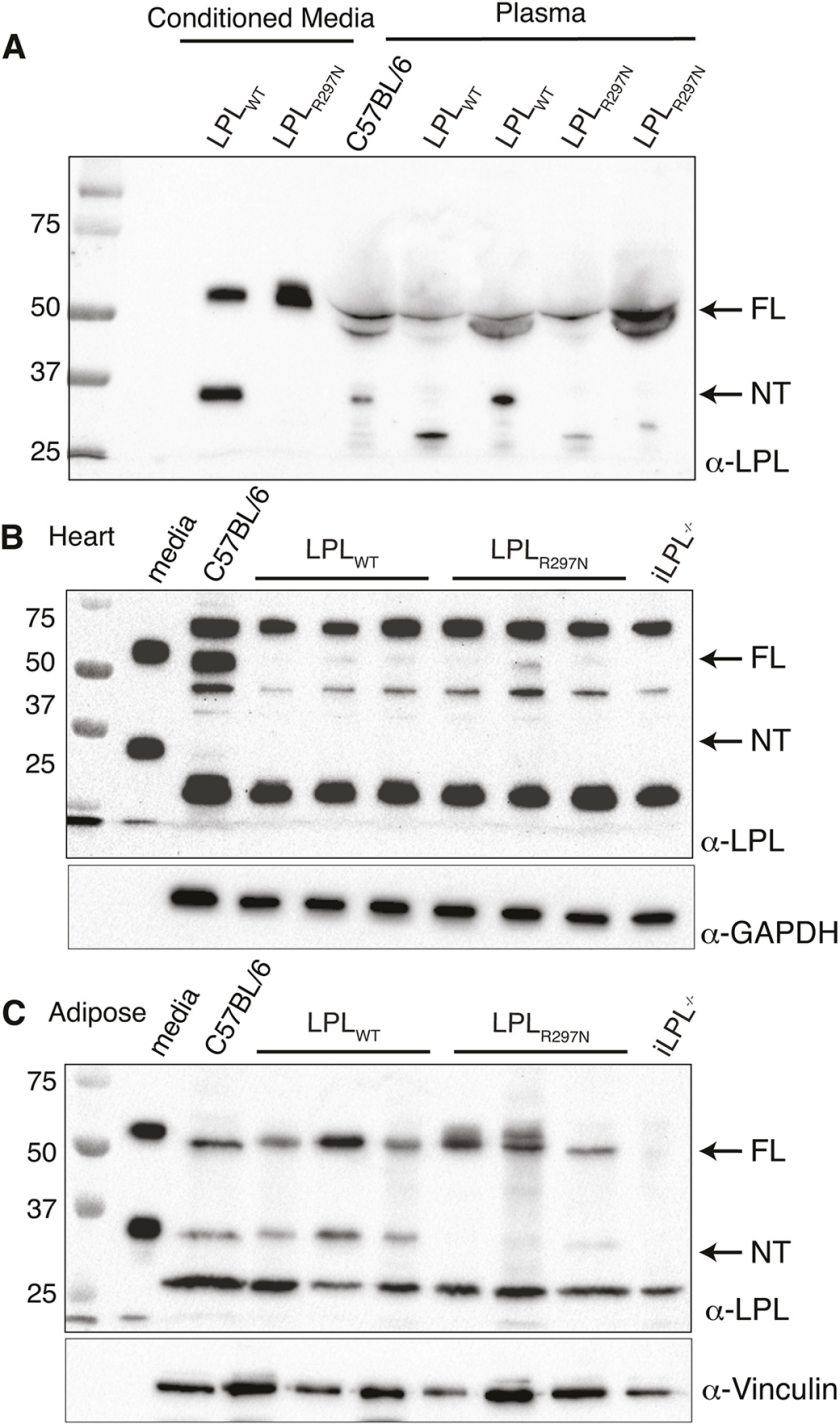
**Furin-mediated cleavage of LPL in mouse blood and tissues.** (A) LPL_WT_ undergoes furin-mediated cleavage in conditioned medium from cultured cells, resulting in both full-length (FL) and N-terminal (NT) cleavage products, whereas LPL_R297N_ does not. Similarly, both FL LPL and NT LPL are present in blood plasma obtained from a C57BL/6 mouse. Both types of LPL were present in the plasma of whole-body inducible LPL knockout (iLpl^−/−^) mice treated with LPL_WT_, but only FL LPL was present in mice treated with LPL_R297N_. (B) In heart tissue, only FL LPL was observed in C57BL/6 mice, mice treated with LPL_WT_ and iLpl^−/−^ mice treated with LPL_R297N_. (C) In adipose tissue, FL LPL and NT products were observed in C57BL/6 mice and in iLpl^−/−^ mice treated with LPL_WT_. Only FL LPL was observed in iLpl^−/−^ mice treated with LPL_R297N_. α-LPL, anti-LPL antibody; α-GAPDH, anti-GAPDH antibody; α-Vinculin, anti-vinculin antibody.

We used western blotting to analyze LPL cleavage in both blood and tissues of treated mice. When we analyzed the blood, iLpl^−/−^ mice treated with LPL_R297N_ showed only FL LPL at 50 kDa, whereas iLpl^−/−^ mice treated with LPL_WT_ showed both FL LPL and the NT cleavage product, which showed a band at slightly below 37 kDa (Fig. 3A), indicating furin-mediated cleavage. We used blood from an untreated C57BL/6 mouse as a source of endogenous LPL, and found both FL LPL and the NT cleavage product present in the blood. We next looked at LPL in heart and adipose tissue. In heart tissue of both LPL_WT_ and LPL_R297N_, only FL LPL was observed (Fig. 3B), which was consistent with our findings for C57BL/6 mice (Fig. 1). Adipose tissue of iLpl^−/−^ mice treated LPL_R297N_ showed only FL LPL, whereas that of mice treated with LPL_WT_ showed FL LPL as well as the NT cleavage product (Fig. 3C). For both tissues, negative-control iLpl^−/−^ mice treated with luciferase via AAV did show non-specific background bands but none for LPL. Thus, LPL_R297N_ is resistant to furin-mediated cleavage *in vivo*, and mice treated with LPL_R297N_ show only FL LPL in the fluids and tissues tested by us.

### Effects of furin-resistant LPL on plasma lipid levels

**Fig. 4.**
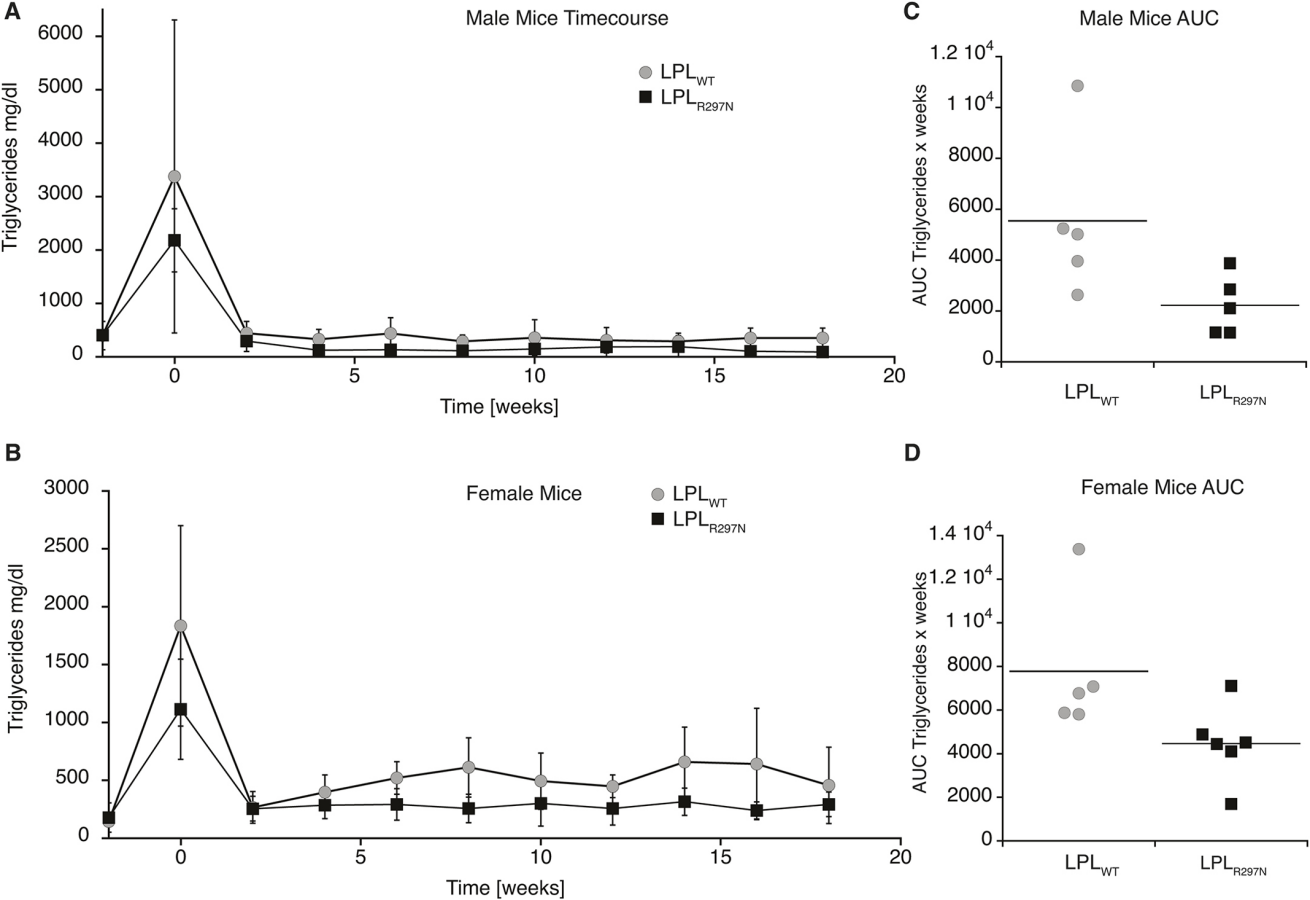
**Plasma triglyceride levels in mice that had been treated with LPL_WT_ or LPL_R297N_.** (A,B) Plasma triglyceride levels in mice treated with LPL_WT_ and LPL_R297N_ were tested every two weeks (week 0−18). Initial levels, at week −2, were low. Mice were then treated with tamoxifen to induce loss of LPL, and triglyceride levels were high when mice were tested at week 0. Mice were treated with LPL_WT_ or LPL_R297N_ at week 0 and plasma triglyceride levels had decreased by week 2. Each data point represents the mean of at least five male and five female mice tested for each condition. Data are the mean±s.e.m. (C,D) To quantify the triglyceride levels over the course of the experiment, the area under the curve (AUC) from week 2 to week 18 was calculated for each mouse. Plots show that average triglyceride levels are lower for LPL_R297N_ than LPL_WT_ in both male and female mice (*P*=0.03 and *P*=0.052, respectively, determined by Wilcoxon rank-sum test).

Having successfully treated LPL-deficient mice with the furin-resistant LPL variant, we next aimed to analyze how furin-mediated cleavage of LPL affects plasma triglyceride levels. Tamoxifen was used to induce LPL deficiency in the iLpl^−/−^ mice and, after two weeks, LPL variants were added back via AAV9-mediated gene transfer. Blood was collected before iLpl^−/−^ mice were treated with AAV; after AAV treatment we collected blood every 2 weeks for the next 18 weeks to compare plasma triglyceride levels of mice treated with LPL_WT_ or LPL_R297N_. Groups of at least five male and five female mice were tested. As shown in Fig. 4A, from week −2 to week 0, triglyceride levels increased upon treatment with tamoxifen and, upon treatment with AAV at week 0, and triglyceride levels decreased by week 2. Triglyceride levels were measure over the next 18 weeks and average levels for both male and female mice are shown in Fig. 4A and B, respectively.

For both male and female mice, triglyceride levels after LPL_R297N_ treatment appeared to be lower than those observed in mice treated with LPL_WT_. To quantify triglyceride levels over the course of the experiment, we measured the area under the curve (AUC) for each individual mouse. We started this measurement at the first timepoint after AAV treatment (week 2). Data for both treatment groups from both male and female mice are presented in Fig. 4C and D, respectively. These data show that triglyceride levels were lower for both the male (*P*=0.03) and female (*P*=0.052) mice in the LPL_R297N_ treatment group relative to the mice in the LPL_WT_ treatment group. These data indicate a significant decrease in triglyceride levels for the male mice and a non-significant lowering trend for the female mice. To ensure that the decrease in plasma triglyceride levels was due to treatment with LPL, we treated iLpl^−/−^ mice with the gene for firefly luciferase delivered via AAV9 as a negative control. As expected, plasma triglyceride levels remained very high, such that mouse survival decreased. As a result, only two male and two female mice were tested with luciferase treatment (see Fig. S1).

**Fig. 5.**
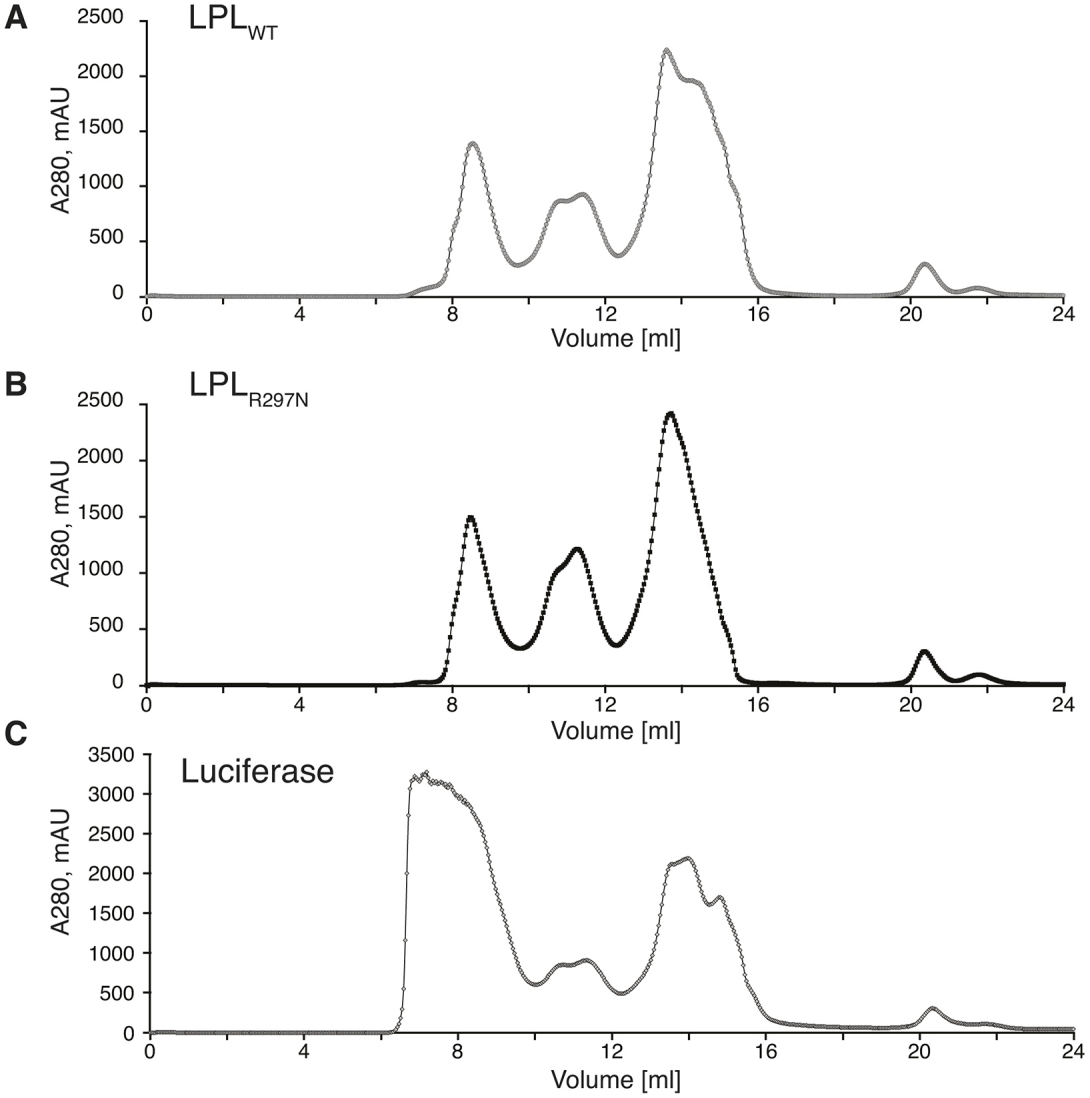
**Lipoprotein profiles for LPL_WT_ vs LPL_R297N_ mice.** At 10 months of age iLpl^−/−^ mice treated with LPL_WT_, LPL_R297N_ or luciferase as a negative control were killed, and blood plasma samples collected and separated on a 24 ml Superose 6 column. (A) Lipoprotein profile of blood from a mouse treated with LPL_WT_. The area under the curve for the three main peaks (from left to right) was 1596, 1709 and 4611 milli-absorbance units (mAu)×ml. (B) Lipoprotein profile of blood from a mouse treated with LPL_R297N_. The area under the curve for the three main peaks (from left to right) was 1632, 1950 and 3783 mAu×ml, respectively. (C) As a negative control, plasma from iLpl^−/−^ mice treated with luciferase showed very high levels of triglyceride rich lipoproteins. The area under the curve for the three main peaks (from left to right) was 7184, 1874 and 4707 mAu×ml, respectively. All profiles are representative of two independent trials.

We also analyzed the lipoprotein profiles of mice in the different treatment groups. Blood was collected at termination, and 200 μl of plasma was applied to a superose 6 gel filtration column. As shown in Fig. 5A-C, lipoprotein profiles appeared similar in the LPL_WT_ and LPL_R297N_ treatment groups. By comparison, negative-control iLpl^−/−^ mice treated with luciferase showed a much higher peak at ∼8 ml, which represents an increase in triglyceride-rich lipoproteins.

### Analysis of tissues

**Fig. 6.**
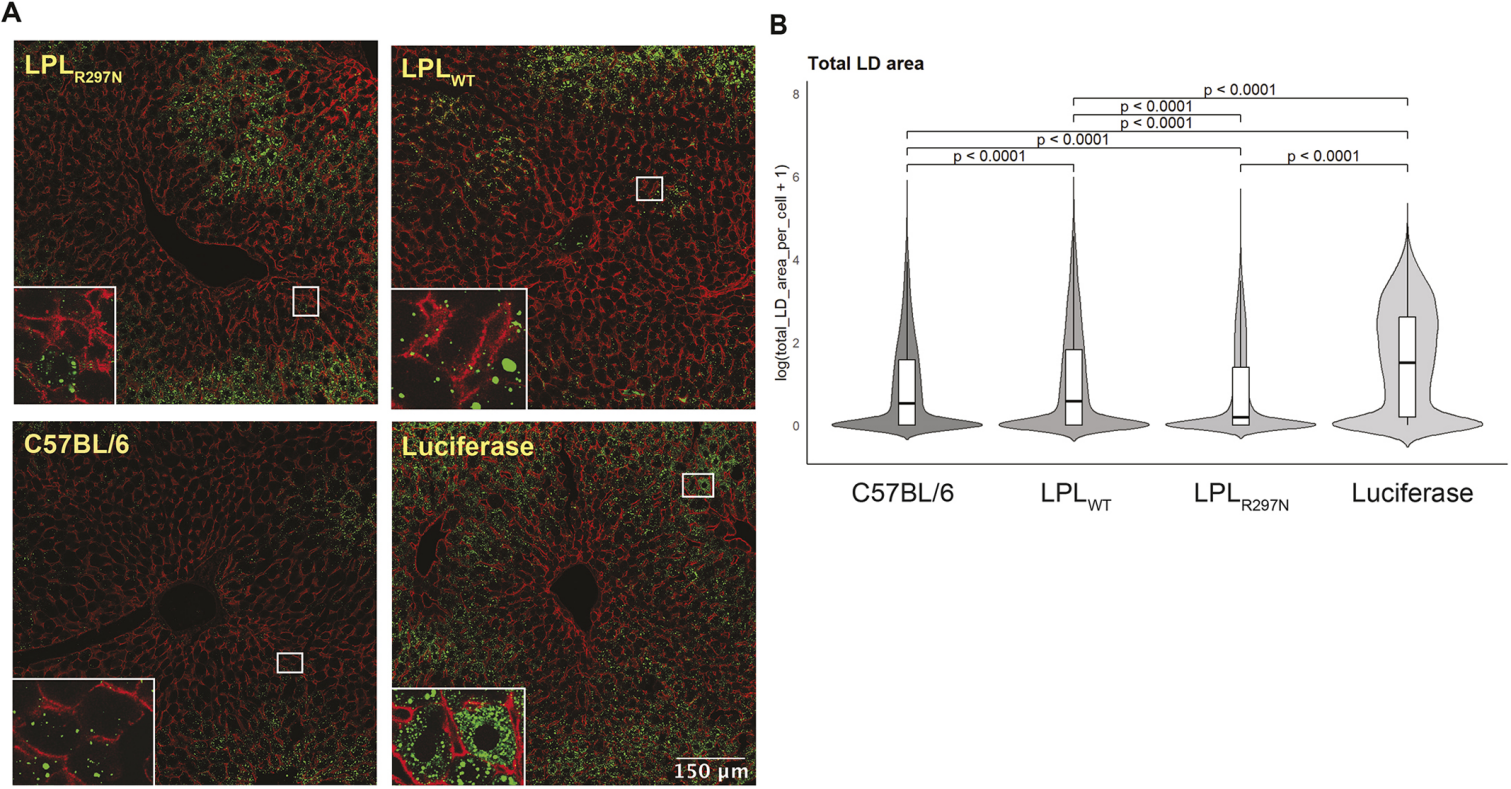
**Liver histology of LPL_WT_ vs LPL_R297N_ mice.** (A) Microscopy images showing representative liver sections from C57BL/6 mice, and iLpl^−/−^ mice treated with LPL_WT_, LPL_R297N_ or luciferase. Samples were stained for lipid droplets using BODIPY(green) and a sodium potassium ATPase antibody was used to mark plasma membrane (red). Imaged sections were centered around the portal vein to select for regions with consistent exposure to plasma triglycerides. Boxed areas are shown magnified (LPL_R297N_, 4.1×; LPL_WT_, 5.4×; C57BL/6, 5.7×; Luciferase, 4.4×) bottom left of each image. (B) Violin plots showing the total lipid droplet (LD) area per cell [µm^2^], calculated using custom Matlab scripts at a log scale [y′=log(y+1)] with medians and IQRs (interquartile ranges). *P* values shown above brackets were calculated using Bonferroni-adjusted Dunn post-hoc tests.

At age 10 months, mice were sacrificed, and blood and heart, adipose and liver tissue were collected. One concern when modulating plasma lipid levels is lipid accumulation in peripheral tissues. To determine if the LPL variants caused lipid accumulation in the periphery, we undertook an examination of liver histology. To quantify the level of lipids in the liver, we embedded and sectioned the liver tissue. Images were centered on the portal vein of the liver to maintain consistency in the imaged area. Liver-cell boundaries were defined by staining against the sodium/potassium-transporting ATPase alpha-1 subunit as a plasma membrane marker. Lipid droplets were stained with BODIPY. Sections were stained, mounted and imaged, and representative liver sections for each condition are shown in Fig. 6. At least three biological replicates were collected per condition and >60 images were analyzed for each LPL variant. Images were analyzed using custom-built MATLAB scripts code that measured the number, area and distribution of droplets.

As shown in Fig. 6B, Kruskal–Wallis test by ranks was used to compare the median of the non-normally distributed populations of segmented lipid droplet area per cell for the C57BL/6 mice, and the iLpl^−/−^ mice treated with LPL_WT_, LPL_R297N_ or luciferase. The calculated *P* value using this test was *P*<0.0001 between each population, indicating different distributions of lipid droplet area per cell with each treatment. Corresponding to measures of triglyceride levels, the median of iLpl^−/−^ mice treated with LPL_R297N_ was found to be the lowest. The negative-control iLpl^−/−^ mice treated with luciferase had the highest lipid droplet area per cell. This indicates that the reduced levels of triglycerides in plasma due to treatment with the LPL variant prevented the lipid buildup in liver tissue as seen in negative-control iLpl^−/−^ mice.

## DISCUSSION

Post-translational modifications are important for regulating the function of enzymes. Members of the proprotein convertase family of proteins, including furin, cause irreversible post-translational modifications, such as proteolytic cleavage. Furin-mediated cleavage separates the catalytic NT domain of LPL from the substrate-binding CT domain, rendering the enzyme inactive (Jin et al., 2005; Liu et al., 2010). Most inquiries into the consequences of furin-mediated LPL cleavage have been undertaken in cultured cells, although one study addressed LPL cleavage through furin in adipose tissue (Dijk et al., 2018). Since furin levels vary considerably by both cell line and tissue type, the outcomes of furin-mediated LPL *in vivo* are unclear [The Human Protein Atlas, 2024 (https://www.proteinatlas.org/ENSG00000140564-FURIN/tissue); Sjostedt et al., 2020]. Here, we not only examined the LPL fragments present in different tissues of WT mice but also directly assessed the effects of furin-mediated cleavage of LPL in a knockout mouse model.

We found different levels of furin-mediated LPL cleavage in different types of tissue. Whereas we saw the expected furin-mediated cleavage of LPL in adipose tissue as well as LPL fragments in plasma (Figs 1 and 3), we did not observe NT cleavage fragments of LPL in kidney, lung, muscle or heart tissue. Rather, these tissues showed more uncleaved LPL. Data from The Human Protein Atlas (2024) indicate that furin expression levels are low in lung, heart muscle, skeletal muscle and adipose tissue (Uhlen et al., 2015). However, furin protein is highly expressed in kidney and liver. Similarly, RNA expression levels of furin are high in the liver but lower heart and skeletal muscle, lung and adipose tissue. Thus, there does not appear to be a strong correlation between furin gene/protein expression levels in tissues and whether FL LPL or LPL cleavage fragments are present.

When we compared the difference in plasma triglyceride levels in iLpl^−/−^ mice treated with AAV delivering either LPL_WT_ or LPL_R297N_, we found that – for both males and females – the LPL_R297N_-treated mice had slightly lower triglyceride levels when levels were integrated over the entire 18-week time course (Fig. 4). These results were significant for the cohort of male mice (*P*=0.03). These data indicate that non-cleavable LPL is slightly advantageous for lowering triglyceride levels in the context of the very high triglyceride levels observed in iLpl^−/−^ mice. Previously published data by Dijk et al. (2018) and our data shown in Figs 1 and 3 indicate that LPL fragments consistent with furin-mediated cleavage are mainly present in plasma and adipose tissue. Taken together, these reduced triglyceride levels suggest that furin-mediated cleavage of LPL does contribute to a decrease in LPL activity *in vivo*.

Prior studies used the human LPL sequence containing the LPL_S447X_ truncation mutation that was beneficial in LPL gene therapy (Hayne et al., 2017; Ross et al., 2005). Our current study indicates that LPL_R297N_ is also a beneficial mutation as it helps to lower plasma triglyceride levels. In iLpl^−/−^ mice treated with either LPL_WT_ or LPL_R297N_, the resulting liver pathology was comparable to that of C57BL/6 mice, effectively avoiding the lipid accumulation observed in the negative-control group treated with luciferase. This indicates that loss of furin-mediated cleavage was not associated with hepatic steatosis. Although clinical gene therapy for patients with familial lipase deficiency is currently unavailable, the potential for future trials or novel LPL-delivery modalities remains. Consequently, ongoing studies aimed at optimizing LPL function for enhanced therapeutic performance remain highly relevant.

## MATERIALS AND METHODS

### Animal studies

All animal studies carried out in this study have been approved by the Institutional Animal Care and Use committee of the University of North Carolina at Chapel Hill under protocol 17-115. C57BL/6 mice with an inducible LPL deletion were generated by crossing mice with a floxed LPL gene (*Lpl^fl/fl^*) (Noh et al., 2006) and mice with beta-actin-driven tamoxifen-inducible Cre (*Mer/Cre/Mer*) to obtain the *Lpl^fl/fl^ Actb3-MerCreMer^+^* offspring, designated as iLpl^−^/^−^ mice (Gordts et al., 2016). Genotype was confirmed by PCR with the following primers for LPL 5′-CGGCTTAGCTAGTAT-3′ and 5′-TCTAGGCAGAGAGCAGCAGA-3′, and for Cre 5′-ATTTGCCTGCATTACCGGTC-3′ and 5′-ATCAACGTTTTGTTTTCGGA-3′. To induce LPL knockout, tamoxifen (Sigma, T5648) was prepared at a concentration of 10 mg/ml in corn oil. Next, 100 μl of tamoxifen reagent was administrated to 8-week-old mice by intraperitoneal (IP) injection for a total of five consecutive days. Successful LPL knockout was assayed by measuring triglyceride levels in the blood, as described for triglyceride assays below. Following LPL knockout, iLpl^−^/^−^ mice were treated with one dose of 1×10^10^ adeno-associated virus (AAV) vector genome copies of per mouse via IP injection. Both male and female mice were tested; no animals were excluded from the analysis.

### Recombinant AAV vector production

AAV was produced using an updated triple-transfection method as previously described (Zolotukhin et al., 1999). Briefly, suspension-adapted human embryonic kidney 293 (HEK293) cells grown to a density of 2×10^6^ cells/ml in FreeStyle F17 expression medium (ThermoFisher Scientific, Cat# A13835-01) were triple transfected with 0.6 μg/ml of pHelper (XX680) plasmid, 0.5 μg/ml of Rep2/Cap and 0.3 μg/ml of the LPL transgene cassette (all obtained from Aravind Asokan, Duke University School of Medicine, Durham, NC, USA; Gonzalez et al., 2023). or the firefly luciferase reporter transgene driven by the chicken beta-actin (CBA) promoter flanked by two inverted terminal repeats (ITR) derived from AAV serotype 2 (AAV2) and complexed with 4.9 μg/ml of PEI MAX (PolySciences, CAS# 49553-93-7). Vectors were purified 6 days post transfection from medium and lysate by using iodixanol-gradient ultracentrifugation followed by buffer exchange into 1× phosphate-buffered saline (PBS) as previously described (Gonzalez et al., 2023). Vector genome titers were obtained by quantitative PCR with primers targeting the inverted terminal repeats (Gonzalez et al., 2023).

### Triglyceride assays

For triglyceride assays, 1 μl of serum was incubated with 40 μl of reagent A [133 mM KPO_4_ pH7.5, 32 μg/ml *Candida Rugosa* lipase (Sigma, L1754) 0.25% fatty-acid-free bovine serum albumin (BSA) for 30 min at 37°C]. Next 50 μl of reagent B [133 mM KPO_4_ pH7.5, 3.3 mM MgCl_2_, 4 mM ATP, 0.6 mM N-ethyl-N-(2-hydroxy-3-sulfopropyl)-m-toluidine (TOOS), 3 mM 4-aminoantipyrine (4-AP), 0.1 mM flavin adenine dinucleotide (FAD), 0.5% Triton X-100, 0.25 U glycerol kinase, 4 U glycerol -3-phospate oxidase, 6 U horseradish peroxidase] was added and samples were incubated for another 50 min at 37°C. Glycerol was used to generate a standard curve for comparison. Samples were put into clear-bottomed 96-well plates and read at 555 nm.

#### Western blotting

LPL present in conditioned medium of cultured cells, prepared as previously described by Wu et al. (2018) served as a positive control. Tissue samples were harvested and proteins extracted using RIPA buffer, and protein levels were quantified using the Pierce BCA Protein assay kit. Next, tissue protein samples (i.e. 30 μg for fat tissue, 50 μg for all other tissue) were suspended in 5×SDS-PAGE loading buffer and separated using SDS-PAGE (10%). Samples were transferred to PVDF membranes, probed with polyclonal goat anti-LPL primary antibody (1:200; R&D systems, cat.# AF7197) and HRP-conjugated donkey anti-goat secondary antibody (1:5000; R&D Systems, cat.# HAF109). Blots were visualized using an HRP kit (Advansta, K-12045). Western blots were imaged using the Bio Rad ChemiDoc MP imaging system.

### Lipoprotein fractionation

The GE Pharmacia AKTA Purifier Fast Performance Liquid Chromatography (FPLC) system was used for fractionation of lipoproteins. For each sample, 200 μl mouse plasma was loaded onto a 24-ml Superose 6 column that had been equilibrated with PBS containing 1 mM EDTA. The system was run at 0.5 ml/min and 1-ml fractions were collected.

### Fluorescence microscopy

Mice were killed aged 10 months, and liver specimens sized ∼1 cm×1 cm collected. Samples were rinsed with 1×PBS, fixed for 24 h in 10% fomalin (neutral buffered), switched to 10% sucrose in PBS for 24 h, followed by 20% sucrose in PBS for the next 24 h and, finally, transferred to 30% sucrose in PBS for 24 h. Samples were embedded in Tissue-Tek O.C.T. Compound (Sakura Finetek, cat.# 4583) and stored at −80° until further use. Sections (5-μm thick) were cut using a cryotome for frozen tissue at −30°C and affixed to Fisher biotech ProbeOn Plus coated slides (Thermo Fisher Scientific). Slides were prepared by incubating with Poly-D-Lysine (0.1% w/v) for 1 h, washed three times with PBS and incubated for 30 min in gelatin solution (1% w/v in PBS).

Tissue sections were blocked in blocking solution (5% BSA, 0.5% Triton X-100) for 35 min at room temperature, then incubated with primary antibody against Sodium Potassium ATPase to stain the plasma membrane in blocking solution (abcam, ab76020, 1:200 in PBS) overnight at 4°C. Primary antibodies were visualized with the corresponding anti-mouse secondary antibody (goat anti-rabbit cross-absorbed secondary antibody, Invitrogen, A11012). Secondary antibodies were conjugated to fluorochrome 555 or 647 (Invitrogen). Sections were washed and subsequently stained with BODIPY to stain lipid droplets at 10% w/v (0.1 mg/ml) for 30 min, and with DAPI at 0.005% w/v (5 μg/ml) to stain nuclei for 10 min, with three washes in between. All data shown are representatives of three or more biological replicates. The number of replicates and images for each sample is shown in supplementary Table S1.

The slides were mounted with ProLong Diamond Antifade Mountant (Invitrogen). Sections were visualized using the Zeiss LSM 710 Spectral Confocal Laser Scanning Microscope with a 40×/1.4 oil Plan Apo objective controlled by Zeiss ZEN 2011 software. Images were deconvolved using the AutoQuant X3 software and cropped using ImageJ FIJI and figures were formatted using Adobe Illustrator. Images were taken centered on the portal vein of the liver lobules to maintain consistency in the imaged area of the lipid lobule. Images were centered on the portal vein rather than the central vein to better examine the changes in areas of lower lipid droplet concentration.

### Image analysis

MATLAB code was written to measure the number, area and distribution of droplets. The code is available in the supplementary information (Supplemental Methods for Analysis of Liver Histology). Lipid droplets were segmented using Niblack's local thresholding algorithm (Niblack, 1986); neighborhood size for the local thresholding was optimized over a range of sizes and comparison to a random set of manually inspected images. The difference between the automatically segmented image and the manually segmented image was measured by using two different methods. Cell areas, which were used in calculating the distribution of lipid droplets area, were segmented using watershed segmentation. Minima used in the watershed segmentation algorithm were determined using Otsu's population-corrected method (Cao et al., 2019) and by identification of branchpoints of skeletonized cell areas, as determined by the built-in skeleton algorithm on MATLAB. Exponential distributions were fitted to the average and total area of lipid droplets per cell for each of the treatments by using GraphPad Prism. Statistical analyses on the mean area of lipid droplets per treatment were done in GraphPad Prism. One-way ANOVA was done with Dunnett's test multiple comparison procedure. Methods of segmentation were chosen by testing various automatic thresholding methods on a random set of experimentally obtained images of varying noise levels and compared to manually segmented versions of the random set.

### Statistics

Descriptive statistics, such as the sample mean and sample standard deviation of triglyceride levels at each time point, were used to draw the line charts by mouse group. The area under the curve (AUC) was calculated using the trapezoidal rule to assess the total triglyceride level over multiple experimental time points for each mouse. The Wilcoxon rank-sum test was used to test whether the mean AUCs between the two groups differ. This test is suitable for comparing two groups when the outcome may be skewed. For image analysis, the total lipid droplet area per cell across four groups (C57BL/6, LPL_WT_, LPL_R297N_, firefly luciferase) was compared using non-parametric tests due to skewed distributions. An overall Kruskal–Wallis test by ranks was used to assess statistical differences between the medians, followed by post-hoc pairwise Dunn's tests with a controlled family-wise error rate. For visualization, violin plots were drawn on a log-transformed scale to reduce skewness. Inferential analyses were not affected by this transformation. We consider a *P*≤0.05 to be statistically significant. We also acknowledge all hypothesis tests yielding large *P* values (*P*≥0.05) as being inconclusive. All statistical procedures were conducted using R 4.3.3 (https://www.r-project.org).

## Supplementary Material

10.1242/dmm.052897_sup1 Supplementary information

## References

[DMM052897C2] Beisiegel, U., Weber, W. and Bengtsson-Olivecrona, G. (1991). Lipoprotein lipase enhances the binding of chylomicrons to low density lipoprotein receptor-related protein. *Proc. Natl. Acad. Sci. USA* 88, 8342-8346. 10.1073/pnas.88.19.8342 1656440 PMC52504

[DMM052897C3] Bish, L. T., Morine, K., Sleeper, M. M., Sanmiguel, J., Wu, D., Gao, G., Wilson, J. M. and Sweeney, H. L. (2008). Adeno-associated virus (AAV) serotype 9 provides global cardiac gene transfer superior to AAV1, AAV6, AAV7, and AAV8 in the mouse and rat. *Hum. Gene. Ther.* 19, 1359-1368. 10.1089/hum.2008.123 18795839 PMC2940566

[DMM052897C4] Cao, X., Li, T., Li, H., Xia, S., Ren, F., Sun, Y. and Xu, X. (2019). A robust parameter-free thresholding method for image segmentation. *IEEE Access* 7, 3448-3458. 10.1109/ACCESS.2018.2889013 31328077 PMC6640864

[DMM052897C5] Chappell, D. A., Fry, G. L., Waknitz, M. A., Muhonen, L. E., Pladet, M. W., Iverius, P. H. and Strickland, D. K. (1993). Lipoprotein lipase induces catabolism of normal triglyceride-rich lipoproteins via the low density lipoprotein receptor-related protein/alpha 2-macroglobulin receptor in vitro. A process facilitated by cell-surface proteoglycans. *J. Biol. Chem.* 268, 14168-14175. 10.1016/S0021-9258(19)85223-7 8314783

[DMM052897C6] Chappell, D. A., Inoue, I., Fry, G. L., Pladet, M. W., Bowen, S. L., Iverius, P. H., Lalouel, J. M. and Strickland, D. K. (1994). Cellular catabolism of normal very low density lipoproteins via the low density lipoprotein receptor-related protein/alpha 2-macroglobulin receptor is induced by the C-terminal domain of lipoprotein lipase. *J. Biol. Chem.* 269, 18001-18006. 10.1016/S0021-9258(17)32409-2 7517936

[DMM052897C7] Dijk, W., Ruppert, P. M. M., Oost, L. J. and Kersten, S. (2018). Angiopoietin-like 4 promotes the intracellular cleavage of lipoprotein lipase by PCSK3/furin in adipocytes. *J. Biol. Chem.* 293, 14134-14145. 10.1074/jbc.RA118.002426 30021841 PMC6130958

[DMM052897C8] Dron, J. S. and Hegele, R. A. (2020). Genetics of hypertriglyceridemia. *Front. Endocrinol. (Lausanne)* 11, 455. 10.3389/fendo.2020.00455 32793115 PMC7393009

[DMM052897C9] Gaudet, D., de Wal, J., Tremblay, K., Dery, S., van Deventer, S., Freidig, A., Brisson, D. and Methot, J. (2010). Review of the clinical development of alipogene tiparvovec gene therapy for lipoprotein lipase deficiency. *Atheroscler. Suppl.* 11, 55-60. 10.1016/j.atherosclerosissup.2010.03.004 20427244

[DMM052897C10] Gaudet, D., Methot, J., Dery, S., Brisson, D., Essiembre, C., Tremblay, G., Tremblay, K., de Wal, J., Twisk, J., van den Bulk, N. et al. (2013). Efficacy and long-term safety of alipogene tiparvovec (AAV1-LPLS447X) gene therapy for lipoprotein lipase deficiency: an open-label trial. *Gene Ther.* 20, 361-369. 10.1038/gt.2012.43 22717743 PMC4956470

[DMM052897C11] Gonzalez, T. J., Mitchell-Dick, A., Blondel, L. O., Fanous, M. M., Hull, J. A., Oh, D. K., Moller-Tank, S., Castellanos Rivera, R. M., Piedrahita, J. A. and Asokan, A. (2023). Structure-guided AAV capsid evolution strategies for enhanced CNS gene delivery. *Nat. Protoc.* 18, 3413-3459. 10.1038/s41596-023-00875-y 37735235

[DMM052897C12] Gordts, P. L., Nock, R., Son, N. H., Ramms, B., Lew, I., Gonzales, J. C., Thacker, B. E., Basu, D., Lee, R. G., Mullick, A. E. et al. (2016). ApoC-III inhibits clearance of triglyceride-rich lipoproteins through LDL family receptors. *J. Clin. Invest.* 126, 2855-2866. 10.1172/JCI86610 27400128 PMC4966320

[DMM052897C13] Hayne, C. K., Lafferty, M. J., Eglinger, B. J., Kane, J. P. and Neher, S. B. (2017). Biochemical analysis of the lipoprotein lipase truncation variant, LPLS447X, reveals increased lipoprotein uptake. *Biochemistry* 56, 525-533. 10.1021/acs.biochem.6b00945 27984852 PMC5848218

[DMM052897C14] Jin, W., Fuki, I. V., Seidah, N. G., Benjannet, S., Glick, J. M. and Rader, D. J. (2005). Proprotein convertases [corrected] are responsible for proteolysis and inactivation of endothelial lipase. *J. Biol. Chem.* 280, 36551-36559. 10.1074/jbc.M502264200 16109723

[DMM052897C15] Jin, N., Matter, W. F., Michael, L. F., Qian, Y., Gheyi, T., Cano, L., Perez, C., Lafuente, C., Broughton, H. B. and Espada, A. (2021). The angiopoietin-like protein 3 and 8 complex interacts with lipoprotein lipase and induces LPL cleavage. *ACS Chem. Biol.* 16, 457-462. 10.1021/acschembio.0c00954 33656326

[DMM052897C16] Kersten, S. (2014). Physiological regulation of lipoprotein lipase. *Biochim. Biophys. Acta* 1841, 919-933. 10.1016/j.bbalip.2014.03.013 24721265

[DMM052897C17] Lafferty, M. J., Bradford, K. C., Erie, D. A. and Neher, S. B. (2013). Angiopoietin-like protein 4 inhibition of lipoprotein lipase: Evidence for reversible complex formation. *J. Biol. Chem.* 288, 28524-28534. 10.1074/jbc.M113.497602 23960078 PMC3789953

[DMM052897C18] Liu, J., Afroza, H., Rader, D. J. and Jin, W. (2010). Angiopoietin-like protein 3 inhibits lipoprotein lipase activity through enhancing its cleavage by proprotein convertases. *J. Biol. Chem.* 285, 27561-27570. 10.1074/jbc.M110.144279 20581395 PMC2934623

[DMM052897C19] Lookene, A., Groot, N. B., Kastelein, J. J., Olivecrona, G. and Bruin, T. (1997). Mutation of tryptophan residues in lipoprotein lipase. Effects on stability, immunoreactivity, and catalytic properties. *J. Biol. Chem.* 272, 766-772. 10.1074/jbc.272.2.766 8995362

[DMM052897C20] Mehta, N., Gilbert, R., Chahal, P. S., Moreno, M. J., Nassoury, N., Coulombe, N., Lytvyn, V., Mercier, M., Fatehi, D., Lin, W. et al. (2023). Preclinical development and characterization of novel adeno-associated viral vectors for the treatment of lipoprotein lipase deficiency. *Hum. Gene. Ther.* 34, 927-946. 10.1089/hum.2023.075 37597209

[DMM052897C21] Niblack, W. (1986). *An Introduction to Digital Image Processing*, Vol. 215. Englewood Cliffs, N.J: Prentice-Hall International.

[DMM052897C22] Noh, H. L., Okajima, K., Molkentin, J. D., Homma, S. and Goldberg, I. J. (2006). Acute lipoprotein lipase deletion in adult mice leads to dyslipidemia and cardiac dysfunction. *Am. J. Physiol. Endocrinol. Metab.* 291, E755-E760. 10.1152/ajpendo.00111.2006 16684851

[DMM052897C23] Peters, R. and Harris, T. (2018). Advances and innovations in haemophilia treatment. *Nat. Rev. Drug Discov.* 17, 493-508. 10.1038/nrd.2018.70 29880919

[DMM052897C24] Ross, C. J., Liu, G., Kuivenhoven, J. A., Twisk, J., Rip, J., van Dop, W., Excoffon, K. J., Lewis, S. M., Kastelein, J. J. and Hayden, M. R. (2005). Complete rescue of lipoprotein lipase-deficient mice by somatic gene transfer of the naturally occurring LPLS447X beneficial mutation. *Arterioscler. Thromb. Vasc. Biol.* 25, 2143-2150. 10.1161/01.ATV.0000176971.27302.b0 16002740

[DMM052897C25] Senior, M. (2017). After Glybera's withdrawal, what's next for gene therapy? *Nat. Biotechnol.* 35, 491-492. 10.1038/nbt0617-491 28591128

[DMM052897C26] Sjostedt, E., Zhong, W., Fagerberg, L., Karlsson, M., Mitsios, N., Adori, C., Oksvold, P., Edfors, F., Limiszewska, A., Hikmet, F. et al. (2020). An atlas of the protein-coding genes in the human, pig, and mouse brain. *Science* 367, eaay5947. 10.1126/science.aay5947 32139519

[DMM052897C27] Stroes, E. S., Nierman, M. C., Meulenberg, J. J., Franssen, R., Twisk, J., Henny, C. P., Maas, M. M., Zwinderman, A. H., Ross, C., Aronica, E. et al. (2008). Intramuscular administration of AAV1-lipoprotein lipase S447X lowers triglycerides in lipoprotein lipase-deficient patients. *Arterioscler. Thromb. Vasc. Biol.* 28, 2303-2304. 10.1161/ATVBAHA.108.175620 18802015

[DMM052897C28] Sukonina, V., Lookene, A., Olivecrona, T. and Olivecrona, G. (2006). Angiopoietin-like protein 4 converts lipoprotein lipase to inactive monomers and modulates lipase activity in adipose tissue. *Proc. Natl. Acad. Sci. USA* 103, 17450-17455. 10.1073/pnas.0604026103 17088546 PMC1859949

[DMM052897C29] Sun, B., Young, S. P., Li, P., Di, C., Brown, T., Salva, M. Z., Li, S., Bird, A., Yan, Z., Auten, R. et al. (2008). Correction of multiple striated muscles in murine Pompe disease through adeno-associated virus-mediated gene therapy. *Mol. Ther.* 16, 1366-1371. 10.1038/mt.2008.133 18560415 PMC2670546

[DMM052897C30] Uhlen, M., Fagerberg, L., Hallstrom, B. M., Lindskog, C., Oksvold, P., Mardinoglu, A., Sivertsson, A., Kampf, C., Sjostedt, E., Asplund, A. et al. (2015). Tissue-based map of the human proteome. *Science* 347, 1260419. 10.1126/science.1260419 25613900

[DMM052897C31] Wu, M. J., Wolska, A., Roberts, B. S., Pearson, E. M., Gutgsell, A. R., Remaley, A. T. and Neher, S. B. (2018). Co-expression of novel furin-resistant LPL variants with LMF1 enhances LPL secretion and activity. *J. Lipid Res.* 59, 2456-2465. 10.1194/jlr.D086793 30318473 PMC6277163

[DMM052897C32] Zhang, R. (2016). The ANGPTL3-4-8 model, a molecular mechanism for triglyceride trafficking. *Open Biol.* 6, 150272. 10.1098/rsob.150272 27053679 PMC4852456

[DMM052897C33] Zolotukhin, S., Byrne, B. J., Mason, E., Zolotukhin, I., Potter, M., Chesnut, K., Summerford, C., Samulski, R. J. and Muzyczka, N. (1999). Recombinant adeno-associated virus purification using novel methods improves infectious titer and yield. *Gene Ther.* 6, 973-985. 10.1038/sj.gt.3300938 10455399

