# Supplementary material for: Functional characterization of furin-mediated lipoprotein lipase cleavage
Source: Dis Model Mech. 2026 Jul 6;19(7):dmm052897. doi: 10.1242/dmm.052897 (PMC13382703; doi:10.1242/dmm.052897)
Supplement: Supplementary information [file dmm-19-052897-s1.pdf]

Figure S1

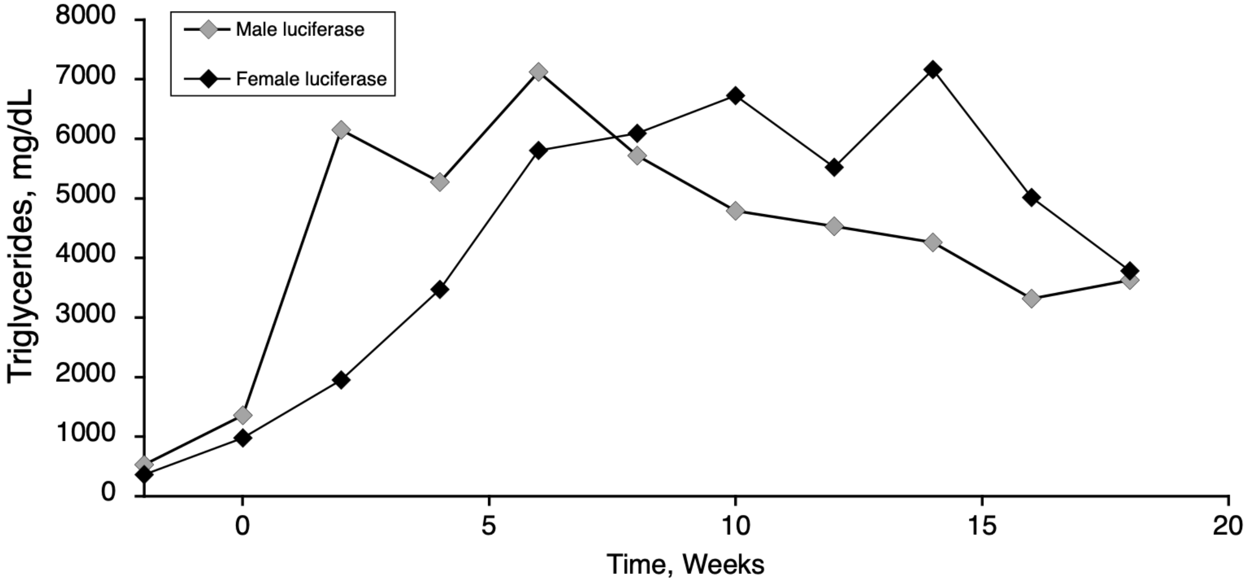

Fig. S1. Plasma triglyceride levels in negative control mice: Plasma triglyceride levels in iLpl$^{-/-}$ mice treated with luciferase were tested every two weeks for 18 weeks. Levels at T=-2, were low, at which time mice were treated with tamoxifen to induce loss of LPL. At T=0, mice were treated with control AAV expressing luciferase and plasma triglyceride levels were measured every two weeks up to 18 weeks. The very high levels of triglycerides reached in these mice were deleterious to mouse survival and data is from two male and two female mice.

Supplemental Methods for Analysis of Liver Histology:

Liver Histology LD segmentation bundle
Last updated 05/6/21
Author: Chelsea Yang

Functions:

cell_seg_smb.m - Runs cell_seg_sm on a folder of .tif image files, creates excel file containing list of detected cells and cell parameters

cell_seg_sm - Runs functions to read the multichannel tiff file, segment the lipid droplets and the membrane, and extract cell and image parameters from the segmented images. Run within the cell_seg_smb function.

Disease Models & Mechanisms • Supplementary information

multichannel_tiff_read.m - Function to read through multichannel .tif files, including z-stack images using the built in Matlab .read function.

droplet_segmentation.m - Extracts lipid droplets from grayscale image using niblick local thresholding.

simple_niblack.m - Locally thresholds image in neighborhoods specified by parameter n.

membrane_segmentation.m - Extracts cell areas from grayscale image using a combination of population corrected Otsu segmentation and watershed segmentation.

pop_corrected_otsu.m - Provides threshold value for grayscale image with a weighting based on population size.

get_cell_loc_sm.m - Extracts specified parameters from cells, saves into an excel file.

Excel sheet modifiers:

image_average_xls.m - Condenses the cell specific information into image specific information, giving total lipid droplet area, total lipid droplet count, and average lipid droplet area per cell.

xls_remove0.m - Removes the 0 values from the excel sheet, removing the data for the cells that contain no lipid droplets. This is not intended to be used on a data sheet where the data has been condensed for the images.

<u>Getting started:</u>

Pre-processing of images: Deconvolve the images. The code works on both cropped and non-cropped images.

Download the cell_seg_smb bundle, add file path to Matlab:

addpath(“file path here”)

Navigate to folder of .tif images to be processed, run

cell_seg_smb(“image folder file path here”)

Make sure to replace the quotes with the full length file path. Ex: ‘/Volumes/biochemistry/final cell_seg bundle’ (Mac). cell_seg_smb will call all of the

other programs in the bundle as needed, other than the scripts intended to modify the saved excel sheets.

The extracted data should be saved in the same folder as the image files after the program finishes running as lipid data.xls. This file will contain a line for each detected cell from each image in the file that gives the total area of lipid droplets within the cell, the average size of lipid droplets within the cell, the number of lipid droplets within the cell, and the distance of each cell from the center of the image. In order to get data for each individual image(combining the parameters pulled from each all cells in an image), use *image_average_xls.m* to get the total lipid droplet area(in pixels), the average lipid droplet area per image, and the total number of lipid droplets in an image. If cells with no lipid droplets must be removed for data analysis or other purposes, use xls_remove0.m to remove cells with no lipid droplets from the data. Both scripts import the existing excel sheet, modify it, and save it under a new name. Row names(image names) are also automatically modified within the script.

Further details on the codes can be found in comments on the individual functions.

```
function cell_seg_smb(fold_path)

%{

Function: cell_seg_smb

Description: Processes a folder of images using Niblack's method for the
segmentation of lipid droplets and the population corrected Otsu method(Cao
et. al, 2019) for the segmentation of cell membranes. The function saves an
excel sheet of data pulled from the processed image and saves it in the
folder indicated by the fold_path parameter.

Parameters: fold_path - file path of folder of images to be processed

Return: No visible return. Function saves excel sheet (.xls) with the
average drop area, total drop area, drop count, and distance to the center
of the image for each cell detected within each image in the indicated file
path.


%}

addpath(fold_path);
files = dir(fold_path);
```

Disease Models & Mechanisms • Supplementary information

```
tot_data = cell(1,size(files,1));

%
%run the single-file processing function cell_seg_sm for each file in the
%directory
%
%data collected from the cell_seg_sm function is collected in a data cell
%and is concatenated at the end of the function run
%

for a = 1:size(files,1)
    if files(a).bytes == 0
        continue
    else

        I = files(a).name;
        if ~strcmp(I(end-3:end),'.tif')
            continue
        else
            [data_cell] = cell_seg_sm(I);
            tot_data{a} = data_cell;
        end
    end
end


%concatenate collected data for processing and saving

tot_data_mat = cat(1,tot_data{:});
count = 0;

for a1 = 1:size(tot_data,2)

    if ~isempty(tot_data{a1})
        count = count + 1;
    end

end

%create cell array to contain image_names for use in the excel sheet
image_names = cell(count,1);
mt = 1;

%populate cell array of image names
```

```
for n = 1:size(files,1)

    [csize,~] = size(tot_data{n});

    if csize == 0
        continue
    end

    name_array = cell(csize,1);

    for n1 = 1:csize
        image_name = files(n).name;
        name_array{n1} = [image_name,'_',num2str(n1)];
    end

    image_names(mt:mt+csize-1) = name_array;
    mt = mt+csize;
end


T = array2table(tot_data_mat,'RowNames',image_names);

write_to = strcat(fold_path,'lipid data.xls');
writetable(T,write_to,'WriteRowNames',true);

end

function data_cell = cell_seg_sm(imagepath)

%{

Function: cell_seg_sm

Description: Single image processing function for liver histology images.
Uses Nickblack's method(neighborhood n = 25) for the segmentation of lipid
droplets and the population corrected Otsu method and watershed
segmentation for the segmentation of cell membranes.

Parameters: imagepath - the file path to the image that is being processed.

Returns: data_cell - a cell array of data, pulled from the image. This
includes the average droplet area, total droplet area, droplet count, and
distance from the center of the image for each detected cell in the image
specified in the input image path.
```

Disease Models & Mechanisms • Supplementary information

```
%}

imstack = multichannel_tiff_read(imagepath);

membrane = imstack(:,:,:,1);
droplets = imstack(:,:,:,2);



%ONLY FOR CROPPED IMAGES: check if selected image is the middle portion of
%the image or the outside of the image. Replace cropped portion of image
%with 0 values if middle of image is cropped out.

max_d = max(max(droplets));
num_max = sum(sum(droplets == max_d));
[a,~] = size(droplets);
perc_max = num_max/a^2;

if perc_max > 0.05
    pos_cropped = droplets == max_d;
    membrane(pos_cropped) = 0;
    droplets(pos_cropped) = 0;
end


%run segmentation methods

drop_thresh = droplet_segmentation(droplets,25);
memb_thresh = membrane_segmentation(membrane);


% pull data from images

data_cell = get_cell_loc_sm(drop_thresh,memb_thresh);

end
```

```
function [drop_thresh] = droplet_segmentation(droplets,nhood)

%{

Function: droplet_segmentation
```

Disease Models & Mechanisms • Supplementary information

Description: Segments the lipid droplets from the green channel as read in
by the multichannel tiff reader. This function is called by cell_seg_sm.
The Niblack local thresholding method is called in simple_niblack in order
to segment the droplets. Automatic neighborhood for the niblack method is
set to n = 25, but can be changed according to size of the lipid droplets;
instructions on testing different neighborhood sizes can be found in the
README.txt. Niblack thresholding method adapted from Jan Motl (2021).
Niblack local thresholding (https://www.mathworks.com/matlabcentral/fileexchange
/40849-niblack-local-thresholding), MATLAB Central File Exchange. Retrieved April 20,
2021.

Parameters:

% droplets - the droplet array, as extracted from the
tiff file. May be a single slice or multiple, if analyzed image is a
z-stack

% nhood - the size of the neighborhood used in the local Niblack thresholding

Returns:

% drop_thresh - binary array in which the lipid droplets are labelled with
value 1 and background is labelled with value 0

%}

```
[~,~,L] = size(droplets);

for a = 1:L
    drip1 = droplets(:,:,a);

    droplet_bw = mat2gray(drip1);
    droplet_bw2 = droplet_bw.*droplet_bw;

    drop_thresh = simple_niblack(droplet_bw2,nhood);
end

end
```

```
function [table] = get_cell_loc_sm(drop_seg,memb_seg)

%{
```

Disease Models & Mechanisms • Supplementary information

Function: get_cell_loc_sm

Description: Extracts various parameters from the processed binary images of membranes and lipid droplets, including average droplet area, total droplet area, droplet count, and distance from center of the image. Data is stored in an array.

Parameters:

% drop_seg - binary array of segmented droplet image, as returned by droplet_segmentation

% memb_seg - binary array of segmented membrane image, as returned by membrane_segmentation

Returns:

% table - An array containing information for each cell detected in the image

%}

```
%check if the segmented membrane image is flipped and correct(membrane
% should be value 1 on a 0 value background)

check_cent = regionprops(memb_seg,'centroid');
[cs1,~] = size(check_cent);

check_flip = regionprops(~memb_seg,'centroid');
[csf,~] = size(check_flip);

if csf>cs1
    memb_seg = ~memb_seg;
end

%clear the border of the image, removing cells that are only partially in
%the image

memb_seg = imclearborder(memb_seg);


%check to see that both the segmented droplet image and segmented membrane
```

```
%image are for the same original tiff file
if sum(size(drop_seg)==size(memb_seg)) ~= 2
    warning('paths for membrane and drop image wrong');
end

lab_memb = bwlabel(memb_seg);
lab_end = max(max(lab_memb));

[r,col] = size(lab_memb);

mid_point = [round(r/2),round(col/2)];

%create zero value arrays to hold the parameters that will be collected for each
%detected cell

drop_count = zeros(lab_end,1);
dropa_avg = zeros(lab_end,1);
dropa_tot = zeros(lab_end,1);
cell_dist = zeros(lab_end,1);



for b = 1:lab_end

    cell_sel = lab_memb == b;

    if sum(sum(drop_seg))/(r*col)>0.9
        drop_seg = ~drop_seg;
    end

    %keep only droplets that are contained inside of detected cells

    drop_sel = cell_sel.*drop_seg;

    tot_area = sum(sum(drop_sel == 1));

    %extract lipid droplet parameters per cell

    lab_drop = bwlabel(drop_sel);
    labd_max = max(max(lab_drop));
    drop_array = zeros(labd_max,1);

    %total droplet area
    for c = 1:labd_max
```

```
        drop_array(c) = sum(sum(lab_drop == c));
    end

    %droplet_count
    drop_count(b) = labd_max;

    %average total droplet area
    dropa_avg(b) = mean(mean(drop_array));
    dropa_tot(b) = tot_area;

    ind_cent = regionprops(cell_sel,'centroid');

    cell_cent = [ind_cent(:,1).Centroid];

    cell_cent_x = cell_cent(1);
    cell_cent_y = cell_cent(2);

    dist_cell = sqrt((cell_cent_x-mid_point(1))^2 + (cell_cent_y-mid_point(2))^2);

    % distance to center
    cell_dist(b) = dist_cell;

end

dropa_avg(isnan(dropa_avg))=0;

%put a identifiable label on each detected cell

label = 1:lab_end;
label = label';
table = [label,drop_count,dropa_avg,dropa_tot,cell_dist];

end
```

```
function image_average_xls(file_name)

%{

Function: image_average_xls.m

Description: Takes the data extracted from cell_seg_smb.m, in which each
row is an individual cell, and condenses it to give all of the data for a
specific image. Each row is an image in the file. Parameters of each image
that are extracted: total droplet area of image(pixels), total droplet
count per image, average area of droplets per cell in image(pixels).
```

Disease Models & Mechanisms • Supplementary information

```
Parameters: file_name - name of excel sheet extracted from cell_seg_smb.m

Returns: No visible returns, but a separate excel sheet is saved in the
same file as the excel sheet that is processed.

%}

cond_table = readtable(file_name,'ReadRowNames',true);

[cond_rnames,cond_array] = mid_out_cond(cond_table);

im_av_dc = zeros(size(cond_array,1),1);
im_tot_dc = zeros(size(cond_array,1),1);
im_av_da = zeros(size(cond_array,1),1);
im_tot_da = zeros(size(cond_array,1),1);


im_av_dc(im_av_dc == 0) = -1;
im_tot_dc(im_tot_dc == 0) = -1;
im_av_da(im_av_da == 0) = -1;
im_tot_da(im_tot_da == 0) = -1;


%setting all of the movethrough elements for moving through the arrays when
%populating
mt = 1;

row_names = cell(size(cond_array,1),1);


%set up inner array of indices, singular one that will used to populate
%all of the actual value arrays
ind_array = zeros(size(cond_rnames,1),1);
ind_mt = 1;

for a = 2:size(cond_rnames)

    %go through all the names
    %if the names match(from same original image), collect indices, add the
    %sums and put into the arrays
    %if the names don't match, move a position and make a new value for
    %collection
```

Disease Models & Mechanisms • Supplementary information

```
%get comparison names
name_prev = cond_rnames{a-1};
name_curr = cond_rnames{a};

tif1_ind = strfind(name_prev,'mid');
tif1_ind_o = strfind(name_prev,'out');

tif2_ind_o = strfind(name_curr,'out');
tif2_ind = strfind(name_curr,'mid');

if isempty(tif1_ind)
    tif1_ind = tif1_ind_o;
end

if isempty(tif2_ind)
    tif2_ind = tif2_ind_o;
end

name_prev = name_prev(1:tif1_ind-1);
name_curr = name_curr(1:tif2_ind-1);

if ~strcmp(name_prev,name_curr)

    %sum up all of the previous stuff
    ind_array(ind_array==0)=[];

    av_dc = mean(cond_array(ind_array,2));
    tot_dc = sum(cond_array(ind_array,2));

    av_da = mean(cond_array(ind_array,4));
    tot_da = sum(cond_array(ind_array,4));

    row_names{mt} = name_prev;
    im_av_dc(mt) = av_dc;
    im_tot_dc(mt) = tot_dc;
    im_av_da(mt) = av_da;
    im_tot_da(mt) = tot_da;

    %switch the elements, create new ind array
    mt = mt + 1;
    ind_array = zeros(size(cond_rnames,1),1);
    ind_mt = 1;

else
```

Disease Models & Mechanisms • Supplementary information

```
        ind_array(ind_mt) = a;
        ind_mt = ind_mt + 1;
    end

end

%remove all empty and 0 values
row_names = row_names(~cellfun('isempty',row_names));

%now condense the mid and out ones
im_av_dc(im_av_dc == -1) = [];
im_tot_dc(im_tot_dc == -1) = [];
im_av_da(im_av_da == -1) = [];
im_tot_da(im_tot_da == -1) = [];

%write into table
T = array2table([im_av_dc,im_tot_dc,im_av_da,im_tot_da],'VariableNames',{'average
drop count','total drop count','Average Drop Area','Total drop
areas'},'RowNames',row_names);
writetable(T,'image cond data 1.xls','WriteRowNames',true);

end

function [mout_names,mout_array] = mid_out_cond(table)

%function to rearrange so that the mid and the out images are right next to each other
and can be added...I shouldn't have to do this but
%here we are

cond_rnames = table.Properties.RowNames;
cond_array = table2array(table);

mout_array = zeros(size(cond_array));
mout_names = cell(size(cond_rnames));

mt = 1;

for a = 1:size(cond_rnames,1)

    if isempty(cond_rnames{a})
        continue
    end

    %theoretically, they should all be mid names
```

```
    mid_name = cond_rnames{a};
    m_index = strfind(mid_name,'mid');
    o_index = strfind(mid_name,'out');

    if isempty(m_index)
        ind = o_index;
    else
        ind = m_index;
    end

    base_name = mid_name(1:ind-1);

    mout_names{mt} = mid_name;
    mout_array(mt,:) = cond_array(a,:);
    mt = mt+1;

    for b = a+1:size(cond_rnames,1)

        if isempty(cond_rnames{b})
            continue
        end

        comp_name = cond_rnames{b};

        if contains(comp_name,base_name)
            mout_names{mt} = comp_name;
            mout_array(mt,:) = cond_array(b,:);
            mt = mt+1;

            cond_rnames{b} = [];
        end

    end


end

end
```

```
function [memb_thresh] = membrane_segmentation(membrane)

%{

Function: membrane_segmentation
```

Disease Models & Mechanisms • Supplementary information

```
Description: Segments the cell membranes using the population corrected
Otsu method and watershed segmentation.

Parameters:

% membrane - grayscale image of membrane layer (works with membrane stain
channel of z-stack image as well)

Returns:

% memb_thresh - thresholded binary image of cell areas

%}

[~,~,L] = size(membrane);

for b = 1:L
    memb_layer = membrane(:,:,b);

    %gaussian filter to get rid of high frequency noise in the image

    gf = imgaussfilt(memb_layer,5);

    %adaptive threshold to get rid of light to dark gradient across image
    %if it exists

    gfbw = mat2gray(gf);
    adj = adaptthresh(gfbw,0);
    gf_adj = gfbw./adj;
    gf_adj(isnan(gf_adj)) = 0;


    %initial thresholding using the population corrected otsu method to
    %generate minima for the watershed segmentation process

    seed_tvalue = pop_corrected_otsu(gf_adj);
    seed_1 = gf_adj>seed_tvalue/2;

    %check if binary image is flipped, correct as needed

    [r,c] = size(seed_1);
    seed_1_rem = imdilate(seed_1,strel('disk',10));
    rem_bw = bwlabel(seed_1_rem);
    del_val = rem_bw(round(r/2),round(c/2));
```

```
if del_val == 0
    seed_1_mask = ones(r,c);
elseif del_val == mode(mode(rem_bw))
    seed_1_mask = ones(r,c);
else
    rem_bw(rem_bw == del_val) = 0;
    rem_bw(rem_bw~=0) = 1;

    %delete the 'smallest' objects on the flipped image
    areas_rem = regionprops(logical(~rem_bw),'Area');
    areas = cat(1,areas_rem.Area);
    m_area = max(areas);

    seed_1_mask = ~bwareafilt(logical(~rem_bw),[m_area-1 inf]);

end


 seed_1 = imdilate(seed_1,strel('disk',3));
 seed_1 = seed_1.*seed_1_mask;
 seed_1_res = seed_1;


 % record positions of objects of a certain size for the identification
 % of seeds while removing connected objects

 num_obj_array = zeros(1,100);
 avg_array = zeros(1,100);
 move_through = 1;
 prev_obj_count = 0;
 obj_count = 3;

 while obj_count > 2
     seed_1 = imdilate(seed_1,strel('disk',3));
     seed_1 = bwmorph(seed_1,'close');

     seed_1bw = bwlabel(~seed_1);

     areas = regionprops(logical(seed_1bw),'Area');
     areas = cat(1,areas.Area);
     avg_array(move_through) = mean(areas);

     obj_count = max(max(seed_1bw));
```

```
        num_obj_array(move_through) = obj_count ;
        move_through = move_through + 1;

        % identifies dilated image with the most separate objects

        if obj_count>prev_obj_count
            seed_fin = seed_1;
            prev_obj_count = obj_count;
        end
    end

    % Preserves objects that are too small to be counted in the dilation
    % process

    small_seed = bwareafilt(~seed_1_res,[0 500]);
    small_seed = imerode(small_seed,strel('disk',1));

    seed_fin = ~seed_fin-~small_seed;


   % watershed the separated objects in order to get general object
   % borders to set as maxima for the watershedding process

   bwd = bwdist(~seed_fin);
   bwater = watershed(bwd);

   bgm = bwater == 0;

   % impose minima and maxima, watershed segmentation

   gmag2 = imimposemin(gf_adj,bgm|~seed_fin);
   ws = watershed(gmag2);

   % generate 2nd set of seeds using the branchpoints of binary image
   % skeletons

   skel = bwmorph(~seed_fin,'skel', inf);
   skel1 = bwareafilt(skel,[0 500]);

   branchp = bwmorph(skel1,'branchpoint');
   seed_2 = imdilate(branchp,strel('disk',10));

   % watershed again with the 2nd set of seeds as minima
```

Disease Models & Mechanisms • Supplementary information

```
gmag3 = imimposemin(gf_adj,bgmlseed_2);
ws1 = watershed(gmag3);

% add together the cells detected through the 2 modes of watershed
% segmentation

ws_el = mode(mode(ws));
ws1_el = mode(mode(ws1));

ws_w = ws~=ws_el & ws~=0;
ws1_w = ws1~=ws1_el & ws1~=0;

% Find area filter parameters

ws_areas = regionprops(ws_w,'area');
ws_areas = cat(1,ws_areas.Area);
ws_areas_av = median(ws_areas);

ws1_areas = regionprops(ws1_w,'area');
ws1_areas = cat(1,ws1_areas.Area);
ws1_areas_av = median(ws1_areas);

% Use area to filter detected cells to get rid of incorrectly segmented
% parts of the image
ws_wf = bwareafilt(logical(ws_w),[ws_areas_av*0.5 ws_areas_av*2.5]);

if ws1_areas_av<ws_areas_av/2
    L_log = logical(ws_wf);
else
    ws1_wf = bwareafilt(logical(ws1_w),[ws1_areas_av*0.5 ws1_areas_av*2.5]);

    % Eccentricity filter to get rid of incorrectly segmented parts of
    % the image

    combo = ws_wf + ws1_wf;
    combo1 = bwpropfilt(logical(combo),'eccentricity',[0 0.85]);

    ws_fin = ws_wf + combo1 == 1;

    L_log = logical(ws_fin+ws_wf);
end

memb_thresh = imfill(L_log,'holes');
```

```
    %imshow(memb_thresh);
end

end
```

```
function imstack = multichannel_tiff_read(filename)

%{

Function: multichannel_tiff_read

Description: Reads multi-channel tiff images into the correct format for
processing in the cell_seg_smb/cell_seg_sm functions. Theoretically able to
process z-stack images as well as single slice images, but this has not
been thoroughly tested.

Parameters: filename - the name of the .tif file that is being read

Return: imstack - the data in a 4 dimensional array, (x y z c)

x - row
y - column
z - place in z-stack
c - color(3 colors per z-stack slice)

%}

tstack = Tiff(filename);

[I,J] = size(tstack.read());
Ka = length(imfinfo(filename))/3;
K = length(imfinfo(filename))/3;

%read through tiff using the built in read function
if Ka<1
    K = length(imfinfo(filename))/2;

    data = zeros(I,J,K,2);
    data(:,:,1,1)  = tstack.read();
    tstack.nextDirectory();
    data(:,:,1,2) = tstack.read();
else
    data = zeros(I,J,K,3);
    data(:,:,1,1)  = tstack.read();
    tstack.nextDirectory();
```

Disease Models & Mechanisms • Supplementary information

```
    data(:,:,1,2) = tstack.read();
    tstack.nextDirectory();
    data(:,:,1,3) = tstack.read();
end


for n = 2:K

    if Ka<1
        tstack.nextDirectory()
        data(:,:,n,1) = tstack.read();
        tstack.nextDirectory()
        data(:,:,n,2) = tstack.read();
    else
        tstack.nextDirectory()
        data(:,:,n,1) = tstack.read();
        tstack.nextDirectory()
        data(:,:,n,2) = tstack.read();
        tstack.nextDirectory()
        data(:,:,n,3) = tstack.read();

    end

end

imstack = data;

end
```

```
function [thresh] = pop_corrected_otsu(image)
%explain this one in the supplementary information... I think I personally
%need it too

%{

Function: pop_corrected_otsu

Description: Uses the automatic thresholding Otsu method with a population
correction weighting method which minimizes the preference for thresholding
values that split the populations of foreground and background evenly, as
detailed in Cao et. al, 2019, doi: 10.1109/ACCESS.2018.2889013.

Parameters:
% image - the array(grayscale) to be automatically thresholded
```

```
Returns:
% thresh - the automatically obtained threshold value for the image, above
which will be segmented to 1(foreground) and below which will be segmented to
0(background)

%}


[N,edges] = histcounts(image);
N(end+1) = 0;
mean_array = N.*edges;

[r,c] = size(image);
m = mean(mean(image));

var2 = 0;

for a = 1:size(N,2)
    wb = sum(N(1:a))/(r*c);
    mb = sum(mean_array(1:a))/sum(N(1:a));

    wf = sum(N(a:end))/(r*c);
    mf = sum(mean_array(a:end))/sum(N(a:end));

    %calculate the variance within the foreground class and background
    %class at the current threshold
    var2_new = (wb*wf)*((mb-mf)^2+(mb-m)^2+(mf-m)^2);

    %if variance is higher than the old variance, replace it and set the
    %new best threshold as the one that was obtained
    if var2_new>var2
        var2 = var2_new;
        thresh = edges(a);
    end
end


end
```

```
function [thresh_im] = simple_niblack(im,ns)

%{

Function: simple_niblack
```

```
Description: Locally thresholds grayscale image using a specified ns x ns
neighborhood

Parameters:

%im - grayscale image to be threhsholded

%ns - size of neighborhood to be used in local thresholding

Returns:

%thresh_im - segmented image (not the threshold value, but the binary
array)

%}

%create average image with square neighborhood as determined by input
mean_filt = fspecial('average', [ns ns]);
mean_im = imfilter(im,mean_filt);

%get standard deviation
mean_square = imfilter(im.^2,mean_filt);
deviation = (mean_square - mean_im.^2).^0.5;

thresh_im = zeros(size(im));


k = -0.1;
offset = -0.1;
%yeah, so setting the offset at 0.1 instead of 0 actually differentiates
%the drops (not sure how this would work for the ones...where it was already working

thresh_im(im> mean_im + k * deviation - offset) = 1;

end
```

```
function xls_remove0(file_name)

%{

Function: xls_remove0

Description: Removes cells with no lipid droplets(0 value for total droplet
area) from the excel sheet and saves a separate excel sheet with the
modified data.
```

Disease Models & Mechanisms • Supplementary information

```
Parameters:

%file_name - name of the excel sheet(.xls or .xlsx) to be modified

Returns:

%no visible returns, but the function saves an excel sheet of .xls format

%}

% excel sheet to be modified is read into matlab as a table

cond_table = readtable(file_name,'ReadRowNames',true);
row_names = cond_table.Properties.RowNames;

cond_array = table2array(cond_table);


% positions where the number of LDs is 0 are recorded and deleted

ind_0 = cond_array(:,2) == 0;

row_names(ind_0) = [];
cond_array(ind_0,:) = [];
row_names = row_names(~cellfun('isempty',row_names));


% modified data is saved as a separate excel sheet

T = array2table(cond_array,'RowNames',row_names,'VariableNames',{'Cell label','Drop
Count','Average Drop Area','Total drop areas','Cell dist'});
writetable(T,'0 value removed data.xls','WriteRowNames',true);


end
```
